# Machine learning applications to predict two-phase flow patterns

**DOI:** 10.7717/peerj-cs.798

**Published:** 2021-11-29

**Authors:** Harold Brayan Arteaga-Arteaga, Alejandro Mora-Rubio, Frank Florez, Nicolas Murcia-Orjuela, Cristhian Eduardo Diaz-Ortega, Simon Orozco-Arias, Melissa delaPava, Mario Alejandro Bravo-Ortíz, Melvin Robinson, Pablo Guillen-Rondon, Reinel Tabares-Soto

**Affiliations:** 1 Department of Electronics and Automation, Universidad Autónoma de Manizales, Manizales, Caldas, Colombia; 2 Department of Computer Science, Universidad Autónoma de Manizales, Manizales, Caldas, Colombia; 3 Department of Systems and Informatics, Universidad de Caldas, Manizales, Caldas, Colombia; 4 College of Science and Engineering, Houston Baptist University, Houston, Texas, United States of America; 5 Department of Computer Science, University of Houston Downtown, Houston, Texas, United States of America; 6 Biomedical and Energy Solutions LLC, Houston, Texas, United States of America

**Keywords:** Flow patterns classification, Machine learning, Deep learning, Extra trees, Feature extraction

## Abstract

Recent advances in artificial intelligence with traditional machine learning algorithms and deep learning architectures solve complex classification problems. This work presents the performance of different artificial intelligence models to classify two-phase flow patterns, showing the best alternatives for this specific classification problem using two-phase flow regimes (liquid and gas) in pipes. Flow patterns are affected by physical variables such as superficial velocity, viscosity, density, and superficial tension. They also depend on the construction characteristics of the pipe, such as the angle of inclination and the diameter. We selected 12 databases (9,029 samples) to train and test machine learning models, considering these variables that influence the flow patterns. The primary dataset is Shoham (1982), containing 5,675 samples with six different flow patterns. An extensive set of metrics validated the results obtained. The most relevant characteristics for training the models using Shoham (1982) dataset are gas and liquid superficial velocities, angle of inclination, and diameter. Regarding the algorithms, the Extra Trees model classifies the flow patterns with the highest degree of fidelity, achieving an accuracy of 98.8%.

## Introduction

Artificial intelligence has benefited many different disciplines. This phenomenon has impacted a wide swath of fields as diverse as clinical treatment, economics, internet security, and fluid transport (Mohanta et al., 2020; Kaieski et al., 2020; Zhao et al., 2020). Fluids with two or more phases in a piping system form the flow patterns, which represent the spatial distribution of the phases involved when they flow in a pipe.

When gases and liquids flow simultaneously in a pipe, the phases can distribute themselves in various configurations depending on many variables. The interface distribution determines the configuration, which results in different flow characteristics (Pereyra et al., 2012). There is no convention in the flow patterns number in two-phase flow due to overlapping and characterization subjectivity, especially in transition zones. Shoham (2006) shows the physical mechanisms and respective models of the different transition boundaries.

**Figure 1:**
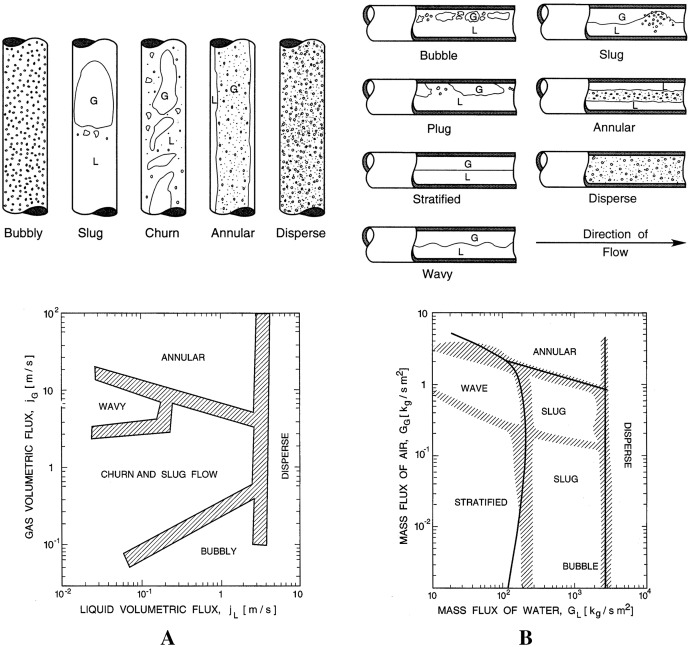
Flow regime map of gas and liquid mixture. (A) Vertical flow. (B) Horizontal flow.

In vertical or moderately deviated pipes, gas-liquid mixtures’ most common flow regimes are the bubble, slug, mist, churn, and annular flow. In horizontal pipes, there may be stratified or wavy stratified flow in addition to many of the regimes observed in vertical wells. Two-phase flow regimes can be represented as plots or maps, using their phase velocities or functions on each axis. Figure 1 shows a flow regime map for a vertical and horizontal flow of a gas and liquid mixture (Brennen, 2005).

Below is the description of each flow pattern as well as its abbreviation:

- Dispersed Bubble Flow (DB). It has the gaseous phase distributed as discrete bubbles within a continuous liquid phase. In other words, the tube carries a specific liquid that has small bubbles evenly distributed over the entire surface. This flow occurs when the rates of liquid are extremely high compared to those of gas.
- Stratified Smooth Flow (SS). It presents the gas flows through the upper part and the liquid through the lower part of the pipe. That is, there is a complete gravitational separation of the two phases. One reason is the higher density of the liquid and the force of gravity exerted on fluids.
- Stratified Wavy Flow (SW). It arises when the velocity of the gas increases in the SS flow, producing large ripples in the liquid. The SS and SW flows are stratified.
- Annular Flow (A). In this regime the gas flows in the center of the tube, as the gas velocity increases, the liquid flows through the tube wall or as scattered droplets.
- Intermittent Flow (I). The distribution of liquid and gas is not uniform. It changes from filling the entire diameter of the tube with liquid to having long spaces with gas. In horizontal flows, plugs or slugs of liquid separated by gas zones fill the whole cross-section of the pipe with a stratified liquid layer flowing along the bottom.
- Bubble Flow (B). It occurs when the gas phase is uniformly distributed and flows as discrete bubbles in a continuous liquid phase. The presence of slip in the bubbles flow allows them to move faster than the liquid phase; that is, there is a relative movement between fluids.

Flow patterns change according to different physical properties and characteristics of the medium. The most notable physical properties of liquid and gas are superficial velocity, viscosity, density, and surface tension. Furthermore, the influence properties of the medium are the angle of inclination and the diameter of the pipe (Paolinelli & Nesic, 2021; Shi et al., 2021; Baba et al., 2020). Given the considerable number of variables, determining the flow pattern is not a trivial task, however, its accurate classification is essential for the proper design and planning of systems with pipelines (Priyanka, Maheswari & Thangavel, 2018; Herrán, de la Cruz & de Andrés, 2010). Multiphase flow is present in different industrial processes with gas or oil pipelines, condensation, or boiling systems, even in nuclear reactor cooling systems (Wang et al., 1990; Ghiaasiaan, 2007; Al-Zaidi, Mahmoud & Karayiannis, 2018; Shi, Cao & Ruan, 2021). They are also present in oil transportation systems where oil is extracted and transported through a network of pipes to processing plants (Bannwart et al., 2004, Al-Safran & Al-Qenae, 2018).

In the study of two-phase flows, the correct estimation is vital due to its relationship with the design variables such as phase holdup, pressure drop, and chemical reaction rate (Pereyra et al., 2012). The present research explores multiple artificial intelligence models for the classification of flow patterns since there is no universal model to perform this task. However, over time, progress has been made in defining the flow pattern, but there is still no theory that accurately characterize flow regimes (Mandhane, Gregory & Aziz, 1974; Barnea et al., 1980; Weisman & Kang, 1981; Barnea, Luninski & Taitel, 1983; McQuillan & Whalley, 1985). Due to the nature of the spatial distribution, there have been disagreements about where a different pattern begins and ends. However, there are flow patterns, and they must be classified correctly (Hernandez et al., 2019; Figueiredo et al., 2020; Liu, Tan & Dong, 2021). Today it is possible to design powerful artificial intelligence models as an excellent tool to predict the flow pattern despite the different changes in the variables involved (Roshani et al., 2021; Cerqueira & Paladino, 2021; de Castro Teixeira Carvalho, de Melo Freire Figueiredo & Serpa, 2020).

In recent years, researchers have developed methodologies to identify multiphase flow regimes. The techniques based on machine learning are fundamental for classification with databases generated in laboratories. The ranking precision they have achieved shows that these methodologies work. The following are some of the studies highlighted for this problem: Xie, Ghiaasiaan & Karrila (2004) uses an Artificial Neural Network (ANN) to classify flow regimes in systems with three phases: gas, liquid, and pulp fiber. Tan, Dong & Wu, 2007 uses the Support Vector Machine (SVM) algorithm to predict the flow regimes using characteristics extracted from electrical resistance tomography data. Ozbayoglu & Yuksel (2012) implemented a neural network model and a regression tree, to identify flow patterns and estimated fluid retention. Al-Naser, Elshafei & Al-Sarkhi (2016) introduces an ANN and data preprocessing with natural logarithmic normalization (which helps reduce overlap between flow patterns), classified the flow patterns. Ezzatabadipour et al. (2017) also highlights recently used deep learning for classification. Furthermore, Amaya-Gomez et al. (2019) proposes a Bayesian supervised learning algorithm with a visualization tool for flow pattern maps. Furthermore, other researchers have presented their results using different models (Azizi, Ahmadloo & Awad, 2016; Guédon, Besagni & Inzoli, 2017).

Previous work on the classification of two-phase flow pattern using the 12 databases to be used in the experiments of this paper reported an accuracy of: 74.71% for the classification of six flow patterns, 79.02% for the classification of five flow patterns, and 82.29% for the classification of three flow patterns, using a mechanistic model (Pereyra et al., 2012); 74.84% for the classification of six flow patterns using a tree-based model (Hernandez et al., 2019); and 95% for the classification of six flow patterns, 95% for the classification of five flow patterns, and 97% for the classification of three flow patterns using a SVM approach (Guillen-Rondon et al., 2018).

This research implements machine learning (ML) techniques in the problem of predicting two-phase flow pattern, it presents machine learning models with their optimized parameters for better classification. This makes possible to determine which are the best alternatives for classifying flow patterns. Extra Trees stands out for its capacity for classification, training, and prediction times, among other models. It was shown that the machine learning techniques implemented are a viable alternative to classify flow patterns where fluids interact in liquid and gas phases. Due to the geometric distribution of the flow patterns, there are combinations of them (Pereyra et al., 2012, Guillen-Rondon et al., 2018); therefore, this work presents the results obtained with these combinations. Since the database is unbalanced to classify flow patterns, techniques are applied to solve the class imbalance, such as random sampling, data sampling and reduction, and other methods like SMOTE and ADASYN. Deep learning architecture designs for this specific problem are presented, showing one of the alternatives for classifying Shoham (1982) data flow patterns and a combination of 12 databases. The metrics used to evaluate the models are cross-validation, accuracy, ROC curves with the area under the ROC curve (AUC) with confidence intervals (CI), precision, recall, f1, confusion matrix, and training curves. These metrics show the effectiveness of the models presented in this work to classify flow patterns.

To summarize, the significant contributions of this paper are the exploration of multiple machine learning models and the identification of the best performing ones to classify flow patterns using two-phase flow regimes (liquid and gas) in pipes. In addition, this work presents a classification system design made up of dataset partitioning, importance and selection of features, hyperparameter adjustment, construction of deep learning architecture, and evaluation of metrics.

The remaining sections of this article are in the following order: “**Materials and Methods**” showing the *Data* and all the extensive treatment done for the problem, including the unions of patterns with similarities, the importance and feature selection, and unbalanced data troubleshooting. The *Models* presents and describes the models used in the investigation. It also shows the *Training model* for the Shoham (1982) data and the 12 databases. The parameters selected for the machine learning models, in addition to the designed neural network architecture. The section also includes *Performance Evaluation*, which shows the metrics used to validate the performance of the model. The section “**Results**” shows the main results obtained for the classification of flow patterns. “**Discussion**” present different analyzes and discusses the results obtained. Finally, the last section presents the “**Conclusions**” of the article.

## Materials and Methods

### Data

The Shoham (1982) database is one of the most significant in the field and it is used in the experiments presented in this paper (Pereyra et al., 2012). This database originated at Tel Aviv University in the first study to consider different angle inclinations. The angle range analyzed went from −90° to 90°, with a mean of 2.727° and a standard deviation of 46.203°. The pipe diameters range used in the experiments went from 25 to 51 mm and have a mean value of 38.657 mm and a standard deviation of 12.985 mm. The data correspond to air-water in atmospheric conditions.

**Figure 2:**
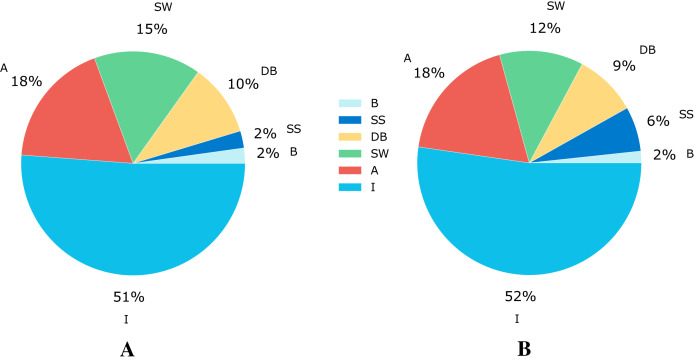
Class distribution for the flow patterns. (A) Data distribution for the Shoham (1982) dataset.(B) Data distribution for 12 DB.

The Shoham (1982) dataset has 5,675 samples, which are categorized in six different flow patterns distributed as shown in Fig. 2A. The number of experiments corresponding to each flow pattern are ‘594’, ‘140’, ‘878’, ‘1,033’, ‘2,905’, and ‘125’ for DB, SS, SW, A, I, and B, respectively.

The flow patterns in the database depend on superficial liquid and gas velocities (Vsl and Vsg), the fluid and gas viscosity (VisL and VisG), the liquid and gas density (DenL and DenG), the surface tension (ST), the angle of inclination of the pipe (Ang), and the pipeline’s diameter (ID).

This paper also involves another dataset, based on Pereyra et al. (2012) and referred in this paper as 12 DB (due to the 12 databases that make up this data set). This complete dataset for flow pattern classification comprises 9,029 samples made up of data from different sources listened in Table 1. Each sample in the dataset is described with nine features (“Vsl”, “Vsg”, “VisL”, “VisG”, “DenL”, “DenG”, “ST”, “Ang”, “ID”) related to operational conditions like superficial liquid and gas velocity, fluid properties such as viscosity, density and surface tension, and pipe configuration parameters like angle and diameter. From the total number of instances, 4,721 correspond to the I flow pattern, 1,664 to A, 1,093 to SW, 816 to DB, 582 to SS, and the remaining 153 to B pattern; this distribution of classes can be seen graphically in Fig. 2B.

**Table 1:** Origin of dataset for 9,029 samples.

Year	Authors	Place
1982	Shoham	Tel Aviv University
1982	Lin	University of Illinois
1986	Kouba	The University of Tulsa
1987	Kokal	University of Alberta
1997	Wilkens	Ohio University
2001	Meng	The University of Tulsa
2001	Manabe	The University of Tulsa
2001	Van Dresar and Siegwarth	NASA
2002	Mata et al.	INTEVEP
2003	Abduvayt et al.	Waseda University
2005	Gokcal	The University of Tulsa
2007	Ombere-Iyari et al.	SINTEF

For further experimentation, the 12 DB produces two new datasets. These are the combination of flow patterns data with similar physical characteristics. The first dataset has a new label named Stratified Flow (ST), which combines instances from labels SS and SW, resulting in five labels. The second dataset has three labels resulting from the combination of Dispersed Flow (DB + B), Segregated Flow (ST + A), and I.

Data preparation has the following operations: assigning each label a numerical value (see Table 2), splitting the data into train and test sets (test set size of 20% like in Guillen-Rondon et al. (2018)), and performing feature standardization using parameters estimated from train data.

**Table 2:** Numeric values assigned to the labels for the twelve datasets.

Flow pattern	Labels		
	6 classes	5 classes	3 classes
SS	1	1	1
SW	2		
A	3	2	
I	4	3	2
B	5	4	0
DB	0	0	

**Figure 3:**
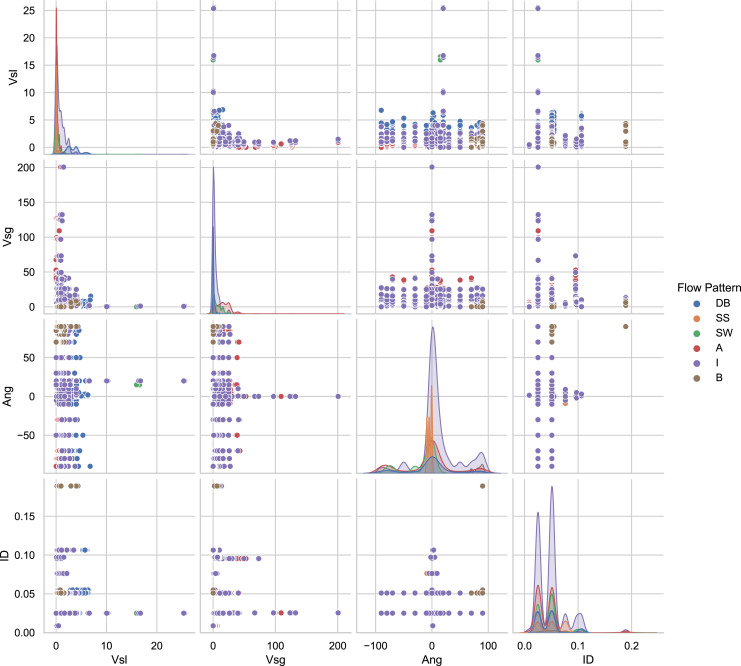
Pair-plot of important input features for flow patterns.

The 12 databases consist of a total of 9,029 experimental data points. Figure 3 presents the distributions of essential variables of the database as an exploratory analysis. For example, in the main diagonal in Fig. 3 is the angle distribution. It demonstrates that the majority of experiments have horizontal pipes. However, some consist of experiments in which pipes are oriented at angles: −90 < *θ* < 90. In Fig. 3 the distribution of pipe diameters is plotted in the main diagonal, showing that the set of data was acquired in 8.74 and 189 mm pipe diameters. The main diagonal of the graph matrix in the Fig. 3 shows the histogram of the superficial gas and liquid velocities. As can be seen, the superficial liquid velocity points are concentrated between 0.00024 and 25.517 m/s. For the superficial gas velocity, the majority of the experiments are between 0.00372 and 200.60976 m/s. Also, a significant number of tests have been carried out in the interval of 0–10 m/s.

Given the nature of the data the human error in some data samples is possible, because each researcher conducting experiments had to make visual observations and judgments to identify each experiment into a flow pattern regime, increasing the possibility of some bias. However, for a given set of pipeline flow conditions, including the operating superficial gas and liquid velocities, fluid properties, and pipe geometry, it is possible to predict the flow pattern.

#### Feature importance and selection

**Figure 4:**
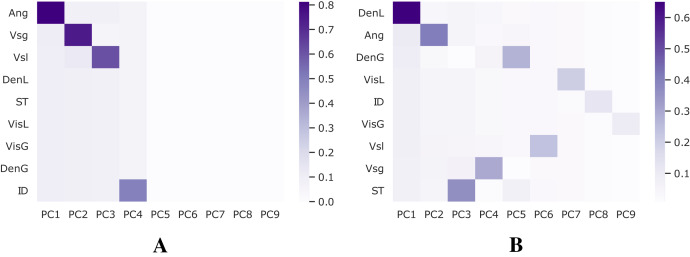
Analysis for feature selection. (A) Shoham (1982) dataset. (B) 12 DB.

A combination of Z-score, also known as standard-score normalization, and Principal Component Analysis (PCA), is applied to the databases employed in this work to compute feature importance and perform feature selection. Keeping the most relevant while eliminating redundant features may decrease noise in the training procedure and improve performance. Figure 4 shows the variance explained for each dataset.

Table 3 shows the results of the feature importance for each dataset. The values are passed by a Softmax function (Reinel et al., 2021), where the sum of all scores is 100% for each dataset.

**Table 3:** Feature importance for Shoham (1982) and 12 DB datasets.

Rank	Shoham		12 DB	
	Feature	Score value (%)	Feature	Score value (%)
1	Ang	16.5	DenL	14.9
2	Vsg	15.5	Ang	12.5
3	Vsl	14.4	ST	11.8
4	ID	12.6	Vsg	10.9
5	DenL	8.2	DenG	10.6
6	DenG	8.2	Vsl	10.5
7	ST	8.2	VisL	10.2
8	VisL	8.2	ID	9.4
9	VisG	8.2	VisG	9.3

Based on these results, only the features Ang, Vsg, Vsl, and ID are selected to train models using the Shoham (1982) dataset since they represent most of its variance. On the other hand, when using the 12 DB dataset, all features are kept.

#### Unbalanced data problem

Addressing the class imbalance problem, Fig. 2 shows a pie chart with the class distribution for the different flow patterns, where 52% of the samples correspond to intermittent, 18% to annular, 12% to stratified wavy, 9% to dispersed bubble, 6% to stratified smooth, and 2% to the bubble flow pattern. The database is not balanced, and it increases the probability of a wrong model generalization. Therefore, we use some techniques to do a class balance improving model learning.

**Figure 5:**
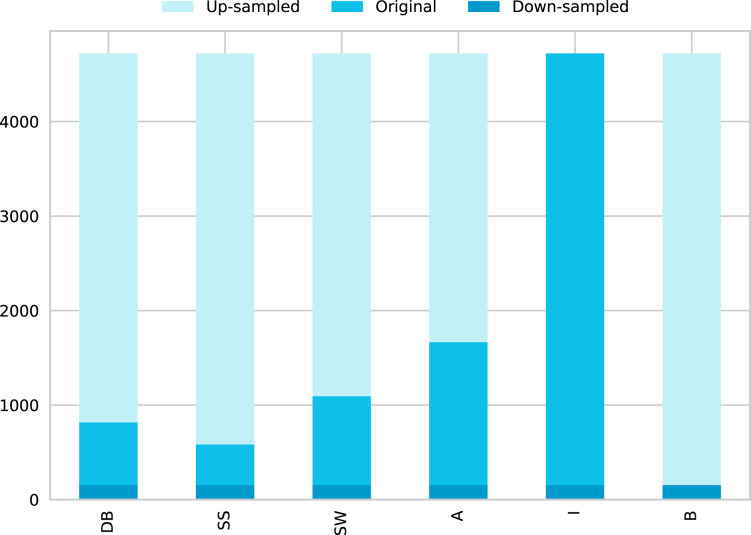
Data distribution by classes for solving unbalanced data using 12 DB. This figure shows three different data distribution (This graph is overlapped, painted in the next order: Up-sampled, Original, and finally Down-sampled).

There are different methods to deal with unbalanced datasets:

*Up-sample Minority Class*. (Figure 5 shows the balanced data using ‘Up-sampling’). Up-sampling refers to the process were training samples from the minority class are randomly duplicated to match the number of samples of the other classes and reinforce its contribution, the most common way of doing so is to resample with replacement (EliteDataScience, 2020).

*Down-sample Majority Class*. (Figure 5 shows the balanced data using ‘Down-sampling’). Down-sampling refers to the process were training samples from the majority class are randomly removed to match the number of samples of the other classes, and level their contribution to the learning algorithm, the most common way of doing so is to resample without replacement (EliteDataScience, 2020).

This paper uses other techniques to solve unbalanced data like *Synthetic Minority Over-sampling Technique (SMOTE)* and *Adaptive Synthetic Sampling Approach for Imbalanced Learning (ADASYN)* to balance the data (Chawla et al., 2002; Haibo et al., 2008). SMOTE creates new samples, which is better than up-sampling that only duplicate existing samples of the minority class, *i.e*., SMOTE synthesizes new samples from the existing samples like data augmentation. ADASYN is like SMOTE, in that it generates synthetic data for the minority classes. However, ADASYN generates more data from samples that are harder to learn from.

### Models

A wide range of recent methods were involved in this project to determine those with the best performance classifying two-phase flow patterns. In addition to selected machine learning algorithms from the python Scikit-Learn library, a deep learning model was designed (Harrington, 2012; Murphy, 2013, Mora-Rubio et al., 2022), using the TensorFlow software which allows the construction of artificial neural networks and convolutional neural networks (Abadi et al., 2015). Although there have been manual mathematical correlation approximations to represent flow patterns, machine and deep learning models have been incorporated to identify them in recent years.

The algorithms analyzed for classification are: Artificial Neural Network (ANN) (Abiodun et al., 2018), Convolutional Neural Network (CNN) (Gu et al., 2018), Extra Trees (ET) (Geurts, Ernst & Wehenkel, 2006), Random Forest (RF) (Breiman, 2001), Gradient Boosting (GB) (Friedman, 2002), Support Vector Machine (SVM) (Platt, 1999; Gholami & Fakhari, 2017), Decision Tree (DT) (Dumont et al., 2009), K-Nearest Neighbors (KNN) (Guo et al., 2003; Zhang, 2016), Quadratic Discriminant Analysis (QDA) (Bose et al., 2015), Gaussian Naive Bayes (GNB) (Pérez, Larrañaga & Inza, 2006) and Ada Boost (AB) (Adaboost et al., 2009).

- **ANN**. ANN, also called multilayer perceptron, defines a mapping *y* = *f*(*x*; *θ*) and learns the values of the parameters, or weights, *θ* that achieve the best approximation from input features *x* to the output *y* (Goodfellow, Bengio & Courville., 2016). An ANN layer consists of several units that compute a linear operation over the input. Then activated by a nonlinear function such as ‘ReLU,’ ‘SeLU’ or ‘TanH,’ this output is passed onto the next layer, successively, until data reaches the output layer; this process is known as the forward pass. The backward pass calculates the prediction error, and the network parameters are updated accordingly to reduce this error. The process of forward followed by a backward pass is repeated for a fixed number of iterations or until convergence (Abiodun et al., 2018).
- **CNN**. It is an extension of the ANN. CNNs have filters that convolve through the data, allowing learning from the training variable’s spatial relationships, often using the pooling operation to reduce dimensionality (Gu et al., 2018). The convolutional layers are connected sequentially, followed by the ANN classification stage, where the last layer has a softmax activation function that allows multiclass classification (Tabares-Soto et al., 2021a).
- **RF and ET**. These models are ensembles of decision trees, an influential and well-known machine learning model for linear classification or regression, but that is prone to overfit the training data (Géron, 2019). To overcome this limitation, RF trains several decision trees, each with a random subset of samples and a subset of features from the whole training set, achieving greater tree diversity, yielding better results (Breiman, 2001). On the other hand, the ET model adds more randomness to the model training process by using random decision thresholds for each feature, rather than searching for the best possible thresholds (Geurts, Ernst & Wehenkel, 2006).
- **GB**. Boosting refers to any Ensemble method that combines several weak learners into a strong learner (Friedman, 2002). The general idea is to train predictors sequentially, each trying to correct its predecessor. GB is a popular boosting algorithm that tries to fit the new predictor to the residual errors made by the previous one (Géron, 2019).
- **SVM**. It solves linear or nonlinear classification and regression. This model performs better for complex small-and-medium-sized datasets. The fundamental idea of classification with SVM is to separate the classes while keeping the decision boundary as far as possible from the closest training samples (Géron, 2019). To add nonlinearity, SVMs uses kernel functions that modify or add features, based on the training set, to create linearly separated classes (Platt, 1999; Gholami & Fakhari, 2017).
- **DT**. This model can perform multiclass classification (Dumont et al., 2009). The algorithm predicts the value of a target variable by learning simple decision rules inferred from the database features. In some cases, it is possible to explain the results with boolean logic. The decision rules become more complex deeper in the tree. Establishing an appropriate depth to the tree is essential to avoid overfitting. This model can be unstable due to variations in the data.
- **KNN**. The model classifies samples making use of the neighboring data points. When evaluating an instance, the model compares it with all data points in the training set. The majority vote of the *K* nearest neighbors of each data provide the corresponding label. The optimal choice of *K* value is highly data-dependent: a larger K suppresses the noise effects but makes classification boundaries less distinct (Guo et al., 2003; Zhang, 2016).
- **QDA**. It is a classifier that uses discriminant analysis to find relations that share two or more features. QDA makes use of nonlinear combinations (Bose et al., 2015). It has a quadratic decision boundary and uses the Bayes’ rule; the model fits a Gaussian density to each class.
- **GNB**. This probabilistic classifier uses Bayes’ theorem (Pérez, Larrañaga & Inza, 2006). The model individualizes each sample feature where no sample will be dependent (assuming the likelihood of features is Gaussian). In other words, each piece can present a probability of belonging to a specific group without the different samples’ presence.
- **AB**. This algorithm begins by fitting a classifier on the original data and then creates copies of the classifiers from the data (Adaboost et al., 2009). The weights of incorrectly classified instances are adjusted. The subsequent classifiers focused on difficult cases, encompassing the dataset features.

#### Hyperparameter tuning

Grid search and K fold cross-validation allow this work to improve the machine learning algorithms. After selecting a range of hyperparameter possible values, the grid search uses different combinations of them. With this process, the model uses some data to train and another to do testing. After that, the best hyperparameters allow saying what the best result is. Also, the cross-validation technique allows dividing the data in K folds, where K-1 allows training the model, and the other part is for testing. Then, the algorithm trains K times using different distributions, changing the test data, and the K results of test data produce the average result. Using this strategy allows searching the optimal hyperparameters (Kanin et al., 2019).

**Figure 6:**
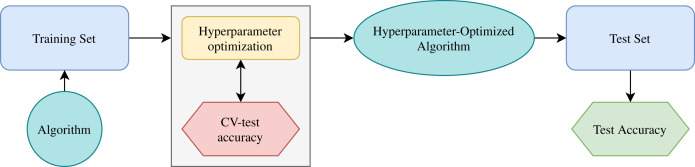
Schematic diagram for working principle of machine learning algorithm.

Figure 6 shows the schematic diagram for the working principle of the machine learning algorithm (Risal et al., 2021). First, the training set evaluates one ML algorithm. Then, the cross-validation splits the training set into data for training and testing. Figure 6 shows in the box the hyperparameter optimization process. There are different combinations of hyperparameters. The CV training data train the algorithm, and the CV testing data generates the accuracy. After this process, test data (never used) allows the final evaluation with the hyperparameter-optimized model. In this work, 10-fold cross-validation allows improving the algorithms to obtain the best performance.

### Model training

#### Shoham (1982) dataset

The first step to find the model with high performance is to determine the best set of hyper-parameters for each one. The hyper-parameter selection is performed training each model with different parameter values and using accuracy metric as a performance indicator. Table 4 shows a set of parameters and ranges (minimum and maximum) to design the ML models and the best values found for each one. A Not Applicable (NA) is placed in the table when the tuned hyper-parameters have no-numerical values. The models have the best performance for this classification problem using the hyper-parameters reported in the last column. These experiments are performed without class balance (original data). The scikit-learn web page explains each parameter in detail (Pedregosa et al., 2011; Buitinck et al., 2013).

**Table 4:** Set of parameters and ranges for designing the ML models and the best values for the Shoham (1982) dataset.

Classifier	Parameter	Minimum value	Maximum value	Best value
**ET**	n_estimators	1	150	112
	min_samples_split	2	100	5
	random_state	1 × 10^6^	100 × 10^6^	28,000,001
**RF**	n_estimators	50	100,000	95
	cc_alpha	1 × 10^−4^	7 × 10^−4^	46 × 10^−5^
	criterion	NA	NA	entropy
	random_state	1	512	277
**GB**	learning_rate	0.01	0.11	0.1
	n_estimators	50	2,150	126
	max_depth	1	10	4
**SVM**	C	1	1 × 10^5^	59 × 10^3^
	gamma	1 × 10^−3^	1	0.0302
**DT**	random_state	1	1,024	1
	min_samples_split	2	2,048	8
**KNN**	n_neighbors	1	100	1
	weights	NA	NA	uniform
	leaf_size	1	100	2
	p	1	2	1
	metric	NA	NA	minkowski
**QDA**	reg_param	1 × 10^−7^	1	786 × 10^−9^
**GNB**	var_smoothing	1 × 10^−9^	500 × 10^−9^	5 × 10 ^9^
**AB**	n_estimator	1	100	6
	learning_rate	0.01	2	0.96
	algorithm	NA	NA	SAMME.R

**Figure 7:**
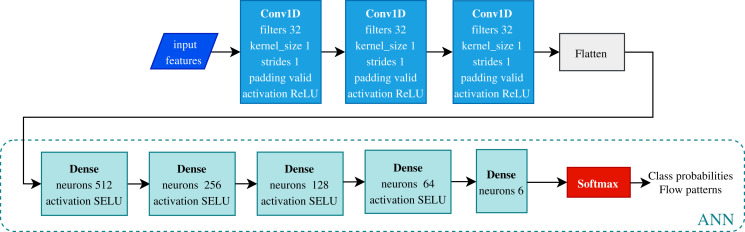
Convolutional neural network architecture. This model classifies six flow patterns using the Shoham (1982) data. The proposed model has a feature extraction stage given by the convolutions and a classification stage using the fully connected neural network (ANN).

Figure 7 shows the network architecture designed and proposed to classify flow patterns. Three layers compose the feature extraction stage, consisting of 1D convolutions with 32 filters of a kernel size of one and ReLU activation. After the pooling layer, a Flatten layer reshapes the outputs to enter the ANN. All fully connected layers use the ‘SELU’ activation function, and the output layer uses a ‘Softmax’ activation function given the multi-class classification task. This CNN has 241,094 total trainable parameters and requires 700 epochs to train with a batch size of 64.

#### 12 DB dataset

The following classifiers use 12 DB: Extra Trees, Support Vector Machine, Random Forest, Gradient Boosting, and a fully connected neural network. The first four models were subject to hyper-parameter tuning to optimize their performance for the task. The parameters and ranges evaluated are presented on Table 5. This table also shows the best parameter for each data configuration. With these parameters, the models have better performance in classifying the flow patterns.

**Table 5:** Set of parameters and ranges for designing the ML models and the best values obtained for 12 DB dataset.

Classifier	Parameter	Range		Best hyper-parameter value		
		Min val	Max val	6 classes	5 classes	3 classes
ET	n_estimators	5	1,000	220	100	100
	min_samples_split	2	5,000	3	3	2
SVM	C	10	3 × 10^6^	120	3 × 10^6^	3 × 10^6^
	gamma	1 × 10^−4^	100	8	0.1	0.5
RF	n_estimators	10	2 × 10^4^	600	1 × 10^4^	140
	criterion	NA	NA	entropy	entropy	entropy
GB	learning_rate	0.01	0.9	0.03	0.03	0.5
	n_estimators	10	9,000	2,048	2,048	2,048
	max_depth	7	1,024	10	7	11

These experiments are different models trained using different balance techniques for the data, like those carried out without class balance. The best models with their hyperparameters are in Table 6. These correspond to Down-sampling, Up-sampling, SMOTE, and ADASYN as data balance techniques.

**Table 6:** Set of parameters and the best values for balancing classes obtained for 12 DB dataset.

Method	Classifier	Parameter	Value	Method	Model	Parameter	Value
**Downsampling**	GB	n_estimators	148	**Upsampling**	ET	n_estimators	100
		max_depth	4			max_depth	42
		random_state	10			random_state	121
	RF	max_depth	22		GB	max_depth	10
		random_state	37			random_state	58
	ET	n_estimators	62			n_estimators	111
		max_depth	32		RF	max_depth	35
		random_state	67			random_state	16
**SMOTE**	ET	n_estimators	42	**ADASYN**	ET	n_estimators	94
		max_depth	None			max_depth	None
		random_state	24			random_state	97
	RF	max_depth	32		RF	max_depth	35
		random_state	83			random_state	18
	GB	max_depth	10		GB	max_depth	9
		random_state	4			random_state	74
		n_estimators	140			n_estimators	120

For the 12 DB the ANN (see Fig. 7) is used for classifying the flow patterns. The network uses 400 epochs and a batch size of 64, RMSprop optimizer, and categorical cross-entropy as the loss function in hyper-parameters training.

### Performance assessment

An important choice when designing artificial intelligence systems is the metrics used to evaluate them (Tabares-Soto et al., 2021b). Below are the metrics used to evaluate the models in this research. Some of them involve the number of predictions of True Positives (TP), True Negatives (TN), False Positives (FP), and False Negatives (FN).

#### Accuracy

The accuracy is the fraction of correct predictions. In some cases, it is represented as a percentage or in values from 0 to 1.

$Accuracy = \displaystyle{{TP + TN} \over {TP + TN + FP + FN}}$

#### Classification report

- **Precision:** it measures a classifier’s ability to identify only the correct instances for each class.

$Precision = \displaystyle{{TP} \over {TP + FP}}$

- **Recall:** also known as sensitivity, it shows the classifier’s ability to find all correct instances by class.

$Recall = \displaystyle{{TP} \over {TP + FN}}$

- **F1:** is a weighted harmonic mean of precision and recall normalized between 0 and 1. F1 score of 1 has a perfect balance due to the relationship between precision and recall. This metric favors unbalanced classes, and it is useful when precision and recall are essential.

$F1 = 2 \times \displaystyle{{Precision \times Recall} \over {Precision + Recall}}$

• **Support:** this metric indicates the number of actual occurrences of the class in test data.

#### ROC curves

Receiver Operating Characteristic (ROC) is used to analyze the output quality of a classifier. This metric shows the balance between classifier sensitivity and specificity, an ideal curve reaching the top left corner indicates high sensitivity and specificity. For multiclass classification, the metric draws each category. And with this curve is associated a vital metric called **AUC**, which for this research incorporates confidence intervals.

#### Confusion matrix

The confusion matrix shows the combination of the actual and predicted classes. Each row of the matrix represents the instances in a predicted class, while each column represents the actual class instances. It allows understanding which labels are most easily confused.

#### Cohen’s kappa

(CK): it is a standard measure to calculate agreement between the classification of qualitative observations. The output is between 0 and 1, the closer to 1, the better is the classifier. Excellent for evaluating multiple classes in unbalanced datasets. The equation represents the expected number of agreements as Pe and the observed as Po.

$CK = \displaystyle{{{P_o} - {P_e}} \over {1 - {P_e}}}$

#### Log loss

(LL): this metric measures the percentage of estimation with the classifier’s negative logarithmic probability.

#### Zero one loss

(ZOL): it is a way of knowing the number of errors in the classification or the error percentage. When the value is zero, it means that the model is working well.

#### Mathews correlation coefficient

(MCC): it has an extension to evaluate multi-class (initially created for binary classification of unbalanced classes) models by computing the correlation coefficient between the actual and predicted labels. A coefficient of 1 represents a perfect prediction.

#### Cross validation

(Cross Valid): it creates different training and testing sets using several partitions of the complete original data and then trains and evaluates a model using each of these partitions.

#### Run time

(RT): this shows the time it took for the algorithm to train and predict.

## Results

The experimental results and the analysis performed in this study are reported in this section. The results include well-structured tables to compare the models, graphs of the training process, classification report and confusion matrix for the best model, and in addition, the section includes ROC curves with CI. The results for the Shoham (1982) database are displayed first and then for the 12 DB. Finally, a comparison of the ROC curves of the databases is presented.

In the experimentation process, the Shoham (1982) dataset (one of the twelve databases of “12 DB”) used 9 ML algorithms in addition to the deep learning architectures. Concerning the results in “12 DB”, 4 ML algorithms and the ANN carried out the experiments. Algorithms for the 12 DB are selected, taking into account the experimental results in the Shoham (1982) database.

### Shoham (1982) dataset

Table 7 presents the result achieved by each model using the Shoham (1982) dataset. Results are sorted by accuracy, with the highest at the top of the table. The best model is ET, followed by RF and SVM for machine learning algorithms.

**Table 7:** Set of metrics for all selected machine learning models.

Model	Accuracy	CK	LL	ZOL	MCC	Cross Valid	RT (s)
ET	0.970	0.956	0.166	0.030	0.956	0.958 *±* 0.013	4.304
RF	0.951	0.927	0.145	0.049	0.927	0.949 *±* 0.007	9.269
SVM	0.948	0.923	0.000	0.052	0.923	0.934 *±* 0.012	32.02
GB	0.944	0.916	0.163	0.056	0.916	0.947 *±* 0.011	56.61
DT	0.934	0.902	1.223	0.066	0.902	0.919 *±* 0.008	0.187
KNN	0.897	0.847	3.560	0.103	0.847	0.887 *±* 0.011	0.325
QDA	0.713	0.582	0.829	0.287	0.589	0.684 *±* 0.028	0.119
GNB	0.695	0.547	0.907	0.305	0.555	0.674 *±* 0.021	0.097
AB	0.670	0.532	1.118	0.330	0.541	0.664 *±* 0.017	0.724

#### Performance of deep learning architecture

**Figure 8:**
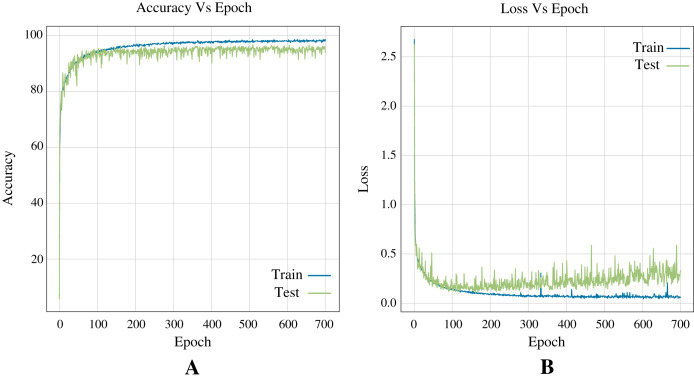
Curves of training process using the CNN model for the Shoham (1982) dataset. This process take 700 epochs to get good models, and each graph shows: (A) Accuracy. (B) Loss.

In terms of deep learning architectures, the CNN outperforms only ANN by accuracy, 96.83%, and 96.39%, respectively. Figure 7 shows the architecture designed. It is important to note that while the only ANN took 1,200 epochs to train, the CNN took 700 epochs. Figure 8 shows the accuracy and loss curves of the CNN’s training process.

#### Performance of the best algorithm

**Figure 9:**
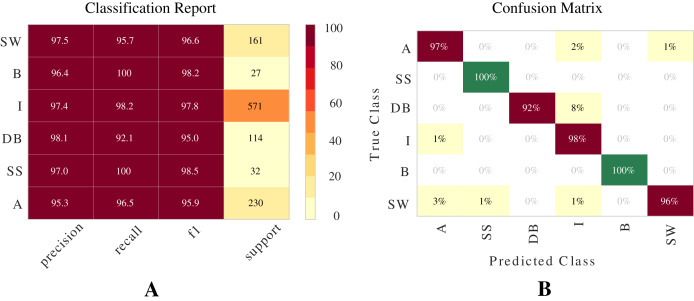
Metrics for Extra Trees model for the Shoham (1982) dataset. These are two important metrics for evaluating models: (A) Classification report. (B) Confusion matrix.

The best model for classifying two-phase flow patterns using the Shoham (1982) dataset is Extra Trees (see Table 7). Figure 9 presents a visual representation of the results using Classification Report and Confusion Matrix. These metrics demonstrate the model’s ability to classify flow patterns.

### 12 DB dataset

As result of the hyper-parameter adjustment process, Table 5 presents the parameter values for each model. Table 8 presents each of the classifiers’ overall performance for 12 DB, like in the Shoham (1982) dataset, the best classifier for the flow patterns classification problem is Extra Trees. The results have the six, five, and three classes defined.

**Table 8:** Model performance.

Model	Cross-validation (acc *±* std) (%)			Accuracy on test (%)			RT (s)
	6 classes	5 classes	3 classes	6 classes	5 classes	3 classes	
ET	94.32 *±* 0.72	94.01 *±* 0.78	95.80 *±* 0.66	94.35	94.68	95.96	27
SVM	92.93 *±* 1.02	92.36 *±* 0.94	94.88 *±* 0.76	92.58	92.80	94.80	5,633
RF	93.98 *±* 1.04	93.65 *±* 0.70	95.51 *±* 0.57	94.13	94.30	95.24	1,634
GB	94.16 *±* 0.82	93.54 *±* 0.61	95.06 *±* 0.46	94.63	94.30	95.35	5,317
ANN	90.86 *±* 1.08	91.48 *±* 1.09	94.10 *±* 1.19	91.25	90.20	92.86	10,649

#### ANN metrics

**Figure 10:**
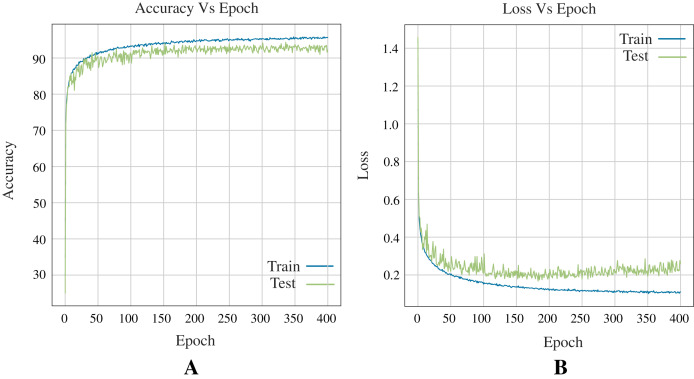
Curves of training process using the ANN model for 12 DB using three classes. This process take 400 epochs to get good models, and each graph shows: (A) Accuracy. (B) Loss.

Figure 10 shows the accuracy and loss curves obtained in training the Neural Network (see Fig. 7) for three classes during 400 epochs.

#### Results balancing data

Tables 9–12 show the results obtained when balancing classes with different metrics. Each class balancing technique shows the best three optimized models for each dataset. The results correspond to the 12 DB for classifying the six flow patterns.

**Table 9:** Set of metrics for results with downsampling data using machine learning models.

Model	Accuracy	CK	LL	ZOL	MCC	Cross valid	RT (s)
GB	0.913	0.895	0.347	0.087	0.896	0.887 *±* 0.042	20.453
RF	0.897	0.876	0.397	0.103	0.876	0.866 *±* 0.041	2.384
ET	0.891	0.869	0.577	0.109	0.870	0.858 *±* 0.033	1.158

**Table 10:** Set of metrics for results with upsampling data using machine learning models.

Model	Accuracy	CK	LL	ZOL	MCC	Cross valid	RT (s)
ET	0.988	0.986	0.055	0.012	0.986	0.987 *±* 0.002	16.454
GB	0.986	0.983	0.049	0.014	0.983	0.986 *±* 0.003	434.065
RF	0.984	0.981	0.061	0.016	0.981	0.985 *±* 0.003	21.972

**Table 11:** Set of metrics for results with over-sampling data using machine learning models with SMOTE.

Model	Accuracy	CK	LL	ZOL	MCC	Cross valid	RT (s)
ET	0.983	0.980	0.080	0.017	0.980	0.981 *±* 0.001	7.777
RF	0.982	0.978	0.085	0.018	0.978	0.979 *±* 0.002	29.881
GB	0.981	0.977	0.077	0.019	0.977	0.979 *±* 0.003	662.904

**Table 12:** Set of metrics for results with over-sampling data using machine learning models with ADASYN.

Model	Accuracy	CK	LL	ZOL	MCC	Cross valid	RT (s)
ET	0.981	0.978	0.081	0.019	0.978	0.981 *±* 0.001	17.376
RF	0.981	0.977	0.081	0.019	0.977	0.979 *±* 0.003	29.562
GB	0.980	0.976	0.071	0.020	0.976	0.980 *±* 0.002	632.558

The experiments presented by this work with balanced data are done only with the 12 DB because this is conformed for the Shoham (1982) data and other 11 databases, as shown in Table 1, corresponding to the 9,029 samples. Therefore, the best three algorithms were chosen using the class balance in the “DB” based on previous experimentation on the Shoham (1982) data and the same “12 DB” data without class balance.

- **Results downsampling data.** Table 9 shows the results obtained.
- **Results upsampling data.** Table 10 shows the results obtained.
- **Results balancing classes (over-sampling) with SMOTE.** Table 11 shows the results obtained.
- **Results balancing classes (over-sampling) with ADASYN.** Table 12 shows the results obtained.

### ROC curves obtained with the confidence interval

**Figure 11:**
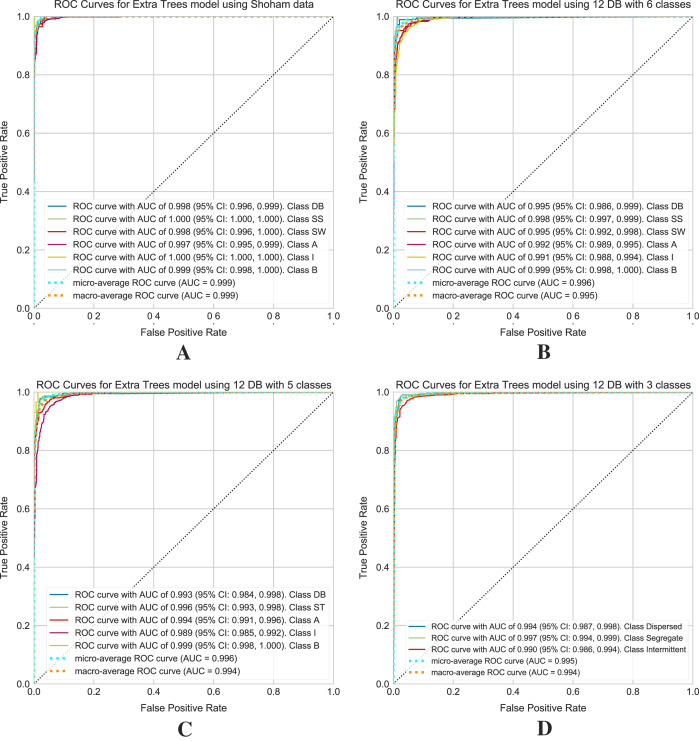
ROC curves for models using the Shoham (1982) and 12 DB, the CI are included for each class. (A) Shoham (1982) dataset. (B) Twelve DB with six classes. (C) Twelve DB with five classes. (D) Twelve DB with three classes.

Figure 11 shows the ROC curves obtained for the selected models on the Shoham (1982) and 12 DB datasets. For 12 DB are the ROC curves with a CI for each class, using six, five and three classes.

## Discussion

This research paper presents the performance of different machine learning models to classify flow patterns, showing the best alternatives for this specific classification problem using two-phase flow regimes (liquid and gas) in pipes.

As part of the classification system design, it can be identified five key aspects: dataset partitioning, feature importance and selection, hyper-parameter tuning, deep learning architecture construction, and metric evaluation. First, partitioning the dataset is an essential data preprocessing step. It is essential to find appropriate data partition sizes that allow the model to generalize well in the training process and demonstrate reliability when evaluated. In this case, the training set represents 80% of the total number of samples and the test set the remaining 20%. Since the adjustment of hyper-parameters was performed using the cross-validation technique, the validation set was not necessary. The feature selection process aims to reduce the dimensions of the dataset without affecting the performance of the model or removing the relevant classification features. A correlation analysis of the features of datasets was performed, reducing the Shoham (1982) dataset to four features (Vsl, Vsg, Ang and ID). They represent 95% of the variability of the data. For the 12 DB dataset, the variability of the data is better distributed among the features. For this reason, it is essential to keep all features.

Regarding the selection of the model and the adjustment of hyper-parameters, an iterative process allowed to find an optimal configuration for each model in a determined evaluation range. Despite the remarkable ability of machine learning models to process and classify data, tuning their hyper-parameters ensures the best performance on its task, as shown in the results presented in this document. The main contribution of this work is the comparison of machine learning models to find the best alternatives. Furthermore, this work presents a deep learning model. It is necessary to find a balance between computational ability, training time, and performance. As a general design rule, a giant network should perform better, but in practice, larger networks take longer to train and tend to overfitting the training set, reducing test set performance. The architecture presented in this article is the product of a meticulous experimentation process, considering aspects such as the number of layers, number of neurons and activation functions. It is leading to remarkable results in classifying flow patterns.

Finally, an extensive set of metrics evaluates the performance of the models. Given the unbalanced nature of the dataset, it is necessary to highlight the F1 score and ROC curves within these metrics to evaluate the classifier. The F1 score is related to two outcomes, precision and recall, and in the case of a multiclass problem, it is necessary to consider the average F1 score of all classes. ROC curves assess the quality of the model and provide a numerical and visual summary of class performance to assess. These curves are useful for analyzing the balance between sensitivity and specificity of the classifier. In this case (multiclass classification problem), the metric draws curves for each category and draws a curve along with its averaged macro and micro-ROC curves. The value to be compared corresponds to the area under the curves, called AUC, with 1 (100%) being the highest value of the ROC curves. The ROC curves with the CI allow the evaluation of the performance of the Extra Trees algorithm. These curves are for Shoham (1982) data and 12 DB with 6, 5, and 3 classes of flow patterns. Based on the Fig. 11A for Shoham (1982) test data is obtained a micro-average ROC curve with AUC of 99.9% and a macro-average ROC curve with AUC of 99.9%, and for 12 DB (see Figs. 11B–11D) is obtained a micro-average ROC curve with AUC of 99.6%, 99.6%, and 99.5%, and a macro-average ROC curve with AUC of 99.5%, 99.4%, 99.4% for 6, 5 and 3 classes, respectively.

The models proposed for the development of this research proved to have distinct levels of effectiveness from one to another. Hyperparameter optimization allows obtaining very accurate models such as Extra Trees with 98.8% with balanced data. However, other models do not achieve good accuracy despite adjusting their hyperparameters, like AB and GNB which did not exceed 67.0% and 69.5%, respectively.

The deep learning models achieve accuracy percentages over 96%, making them slightly less effective than the ET model. The DL architectures need more time to train and predict (250 s for CNN and 264 for ANN, remembering that CNN uses 700 epochs and ANN uses 1,200). The ML models do not waste excessive time; GB is the slowest with 56.6 s. The Extra Trees model mentioned above stands out from all others because it differs from both accuracy and runtime, with an accuracy of 97% and a runtime of only 4.3 s (The runtime includes training time and predicting labels for the test set), using Shoham (1982) dataset. As shown by the results, the Extra Trees model is the most effective classification method for this task and recommended for flow pattern prediction.

One possible limitation of the current work comes from the nature of the data. There could be some bias in the original data because each researcher conducting experiments had to make visual observations and judgments to classify each experiment into a flow pattern regime. As a result, human error is possible in some samples of the data.

## Conclusions

A novel data analytics approach using machine learning is proposed to quantify the prediction of gas-liquid two-phase patterns in pipes. This is carried out by comparing the prediction of available methods against a comprehensive data base collected from the literature, which includes 9,029 data points.

Using an optimized Extra Trees for the classification of flow patterns carries this out. It was shown that the Extra Trees algorithm could learn surprisingly well, as using our hyperparameters tuning allowed us to achieve high precision predicting different combinations of classes.

The 12 databases consolidated in the experiments of this paper are among the largest in the literature for the classification of gas-liquid flow patterns, from previous studies for the classification of flow patterns it can be observed that the results presented in this paper provide novel techniques that can be used in the design of powerful artificial intelligence models as an excellent tool to predict the flow patterns in different industrial processes.

As future work, the methodology designed and presented in this paper can be used in a more significant amount of experimental data, representing even better the conditions of the means of application.

## Additional Information and Declarations

### Data Availability

The following information was supplied regarding data availability: The code and data are available at GitHub: https://github.com/BioAITeam/Machine-learning-applications-to-predict-two-phase-flow-patterns.
